# *Sesuvium portulacastrum*-Mediated Removal of Nitrogen and Phosphorus Affected by Sulfadiazine in Aquaculture Wastewater

**DOI:** 10.3390/antibiotics11010068

**Published:** 2022-01-07

**Authors:** Chaoyue Zhang, Dan Wang, Weihong He, Hong Liu, Jianjun Chen, Xiangying Wei, Jingli Mu

**Affiliations:** 1 Institute of Oceanography, College of Geography and Oceanography, Minjiang University, Fuzhou 350108, China; 3190831040@fafu.edu.cn (C.Z.); wangdanmju@mju.edu.cn (D.W.); heweihong@mju.edu.cn (W.H.); 2 College Resources and Environment, Fujian Agriculture and Forestry University, Fuzhou 350002, China; 000q050020@fafu.edu.cn; 3 Environmental Horticulture Department, Mid-Florida Research and Education Center, Institute of Food and Agricultural Science, University of Florida, 2725 Binion Road, Apopka, FL 32703, USA; jjchen@ufl.edu

**Keywords:** aquaculture wastewater, nitrogen, phosphors, rhizosphere microorganisms, *Sesuvium portulacastrum*, sulfadiazine

## Abstract

Plant-based removal of nitrogen (N) and phosphorus (P) from water bodies is an important method for remediation of aquaculture wastewater. In order to acquire knowledge as to how antibiotic residues in wastewater might affect the microbial community and plant uptake of N and P, this study investigated N and P removal by a coastal plant *Sesuvium portulacastrum* L. grown in aquaculture wastewater treated with 0, 1, 5, or 50 mg/L sulfonamide antibiotics (sulfadiazine, SD) for 28 days and compared the microbial community structure between the water and rhizosphere. Results showed that SD significantly decreased N removal rates from 87.5% to 22.1% and total P removal rates from 99.6% to 85.5%. Plant fresh weights, root numbers, and moisture contents as well as activities of some enzymes in leaves were also reduced. SD changed the microbial community structure in water, but the microbial community structure in the rhizosphere was less affected by SD. The microbial diversity in water was higher than that in the rhizosphere, indicating microbial community differences. Our results showed that the commonly used antibiotic, SD, in aquaculture can inhibit plant growth, change the structure of microbial community, and reduce the capacity of *S. portulacastrum* plants to remove N and P from wastewater, and also raised alarm about detrimental effects of antibiotic residues in phytoremediation of wastewater.

## 1. Introduction

Eutrophication is a serious environmental problem and is expected to increase by 20% to 100% in 2050 and by 120% to 390% in 2100 as per the climate and population forecasts [1]. The discharge of aquaculture wastewater is one of the main sources of eutrophication. Intensive fishing and excess bait supply, along with the presence of fish excreta, result in the pollution of aquaculture water [2,3]. High levels of nitrogen (N) and phosphorus (P) can lead to eutrophication and cause potential harm to the environment and aquatic ecosystems [4,5]. Therefore, it is crucial to remove N and P and prevent the occurrence of eutrophication during aquaculture. Several methods have been used for the removal of pollutants from water. Compared with the traditional physical and chemical remediation techniques, phytoremediation is currently recognized as a low-cost and environmentally friendly method. Increasing evidence shows that plants can take up and utilize the excess N and P from water for their growth and maintain the stability of aquatic ecosystems [6,7]. In addition to plant absorption and utilization, and rhizosphere microorganisms also play a vital role in remediation of water [8]. However, the uncontrolled use of antibiotics during aquaculture may interfere with the phytoremediation process, resulting in poor removal efficiency of N and P.

Sulfonamide antibiotics are one of the most widely used antibiotics in aquaculture, and they have been commonly detected in wastewater [9,10]. This group of antibiotics possess broad-spectrum activity against a wide range of Gram-positive and Gram-negative bacteria and can be used to prevent and treat infectious diseases. Recently, there have been increasing concerns over the residues of sulfonamide antibiotics in aquaculture wastewater [11,12]. The concentrations of sulfonamide antibiotics per liter of wastewater range mostly from nanogram to microgram levels. However, with the continuing use of these antibiotics, the residual concentrations of these antibiotics can reach milligram levels [13,14,15], which can be potentially dangerous to the aquatic environment and can also adversely affect phytoremediation. Sulfadiazine (SD) is a sulfonamide antibiotic whose presence was reported to hamper phytoremediation and restrain plant growth [16]. However, the effects of sulfonamide antibiotics on the process of plant denitrification and P removal and on microbial community have not been well studied.

*Sesuvium portulacastrum* L., a member of the family Aizoaceae, is a perennial succulent halophytic herb. It has high adaptability to adverse environments including tolerance to heavy metals and salinity [17]. It is propagated mainly through cuttings, and cuttings can be readily established in natural conditions. Plants can grow perennially in seashores, saline soils, and wastewater. Previous studies showed that *S. portulacastrum* can be used for bioremediation of heavy metals, N, P, and organic pollutants from water bodies [18,19,20]. As a result, *S. portulacastrum* has been considered as an important plant for phytoremediation.

This study was intended to (1) investigate the effects of different concentrations of SD in wastewater on *S. portulacastrum* absorption of N and P; (2) analyze the microbial community structure in the water and rhizosphere influenced by the increased levels of SD, and (3) gain a better understanding of plant–microbe interactions in the removal of N and P from aquaculture wastewater.

## 2. Results

### 2.1. Effects of Different Concentrations of SD on S. portulacastrum-Mediated Removal of N and P

**Figure 1:**
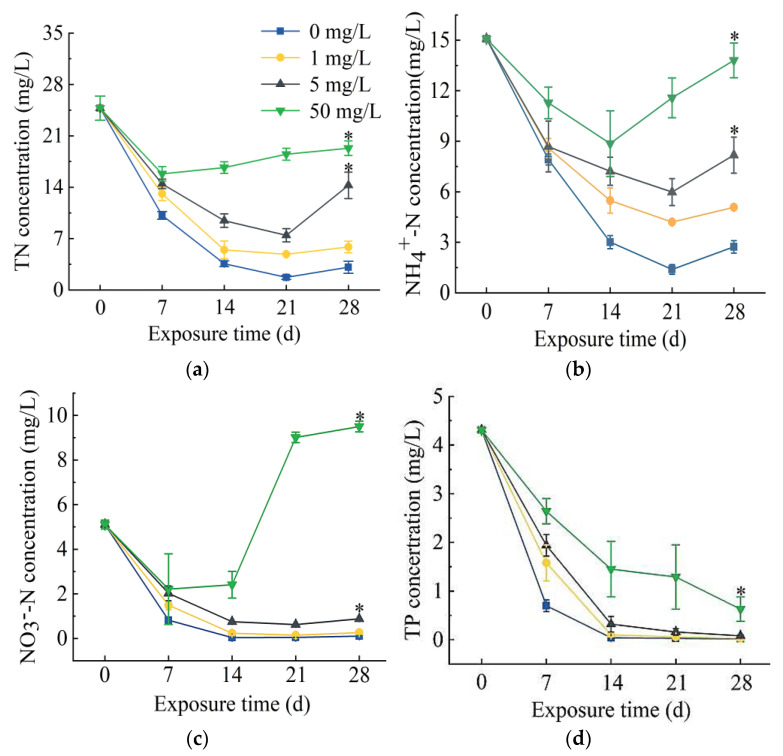
The changes in concentrations of total N (TN) (**a**), NH_4_^+^-N (**b**), NO_3_^–^-N (**c**), and total phosphorus (TP) (**d**) in wastewater treated with 0, 1, 5, or 50 mg/L SD over the course of *S. portulacastrum* plant growth for 28 days. Data are means ± standard deviation (*n* = 3). The asterisk (*) indicates significant differences based on Duncan’s Multiple Range test at *p* < 0.05 level.

In the process of *S. portulacastrum*-mediated removal of N and P, there was a decrease in total N (TN), ammonia N (NH_4_^+^-N), and nitrate N (NO_3_^−^-N) concentrations initially, followed by an increase in their concentrations (Figure 1a–c). After 21 days of exposure, the TN removal rates in 0, 1, and 5 mg/L SD treatments were 93.1%, 80.4%, and 69.9%, respectively. However, the TN removal rate in treatment of 50 mg/L SD was only 36.1%. The removal rate of the control group was the highest among all treatments. The removal rates of NH_4_^+^-N were the highest after 21 days of exposure to 0, 1, or 5 mg/L SD, which were 90.8%, 72.2%, and 60.3%, respectively, while the highest removal rate from 50 mg/L SD treatment was 41.3% on day 14. Again, the removal rates of all SD treatments were significantly lower than the control treatment. The removal rates of NO_3_^−^-N showed similar trends as those of TN and NH_4_^+^-N. The highest removal rates occurred after 21 days of exposure to 0, 1, and 5 mg/L SD, which were 99.1%, 97.1%, and 87.8%, respectively. However, NO_3_^−^-N concentration in 50 mg/L SD treatment started to increase from day 14 and reached 9.5 mg/L on day 28. Total P (TP) concentrations generally showed a decreasing trend (Figure 1d). The highest TP removal rate in the control group was 99.6% after exposure for 28 days, and the TP removal rates in 0, 1, and 5 mg/L SD treatments were 99.4%, 98.1%, and 85.5%, respectively.

### 2.2. Effects of SD on Plant Growth and Physiological Responses

#### 2.2.1. Growth Responses of *S. portulacastrum*

**Figure 2:**
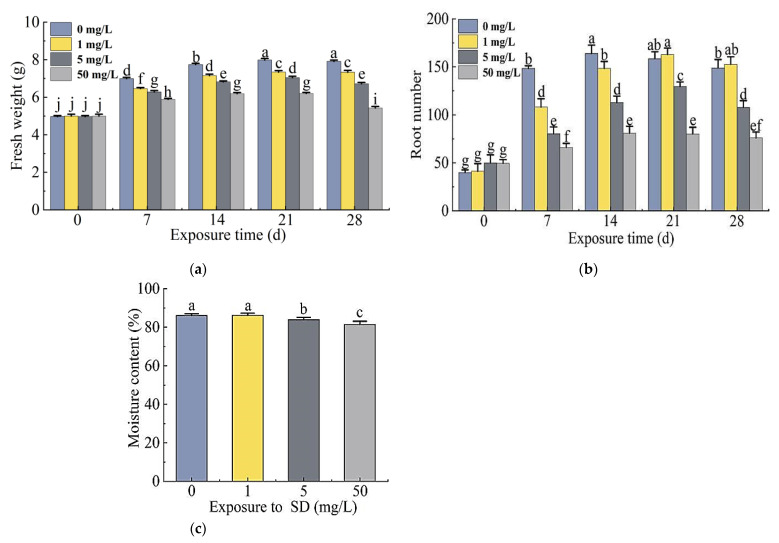
Differences in fresh weights (**a**), root numbers (**b**), and moisture content (**c**) of *S. portulacastrum* plants grown in wastewater treated with 0, 1, 5, or 50 mg/L SD for 28 days. Data are means ± standard deviation (*n* = 3). Different letters above bars represent significant differences based on Duncan’s Multiple Range test at *p* < 0.05 level.

The fresh weight of the plants treated with 0, 1, 5, or 50 mg/L SD increased significantly with the exposure time and reached the highest at 21 days. The fresh weight of the plants treated with 5 mg/L and 50 mg/L SD decreased significantly on day 28. The fresh weight of plants in the SD treatment group was significantly lower than that of the control in each week of exposure except for day 0 (Figure 2a).

The changes in the root numbers of plants in the treatment and control groups were consistent with the fresh weight of the plants, and the highest number of roots occurred in 21 days. On day 28, root numbers of the plants treated with 5 mg/L and 50 mg/L SD decreased significantly compared to the control, while root numbers of plants treated with 1 mg/L SD were higher than those of the control group (Figure 2b).

The moisture content of plants gradually decreased with increased SD concentrations, and the moisture content of plants exposed to 50 mg/L SD was the lowest at 81.4%. There was no significant difference in moisture content between 1 mg/L SD treatment and the control, but the moisture content of plants exposed to 5 mg/L and 50 mg/L SD treatments decreased significantly (Figure 2c).

#### 2.2.2. Physiological Responses of *S. portulacastrum* to SD Stress

**Figure 3:**
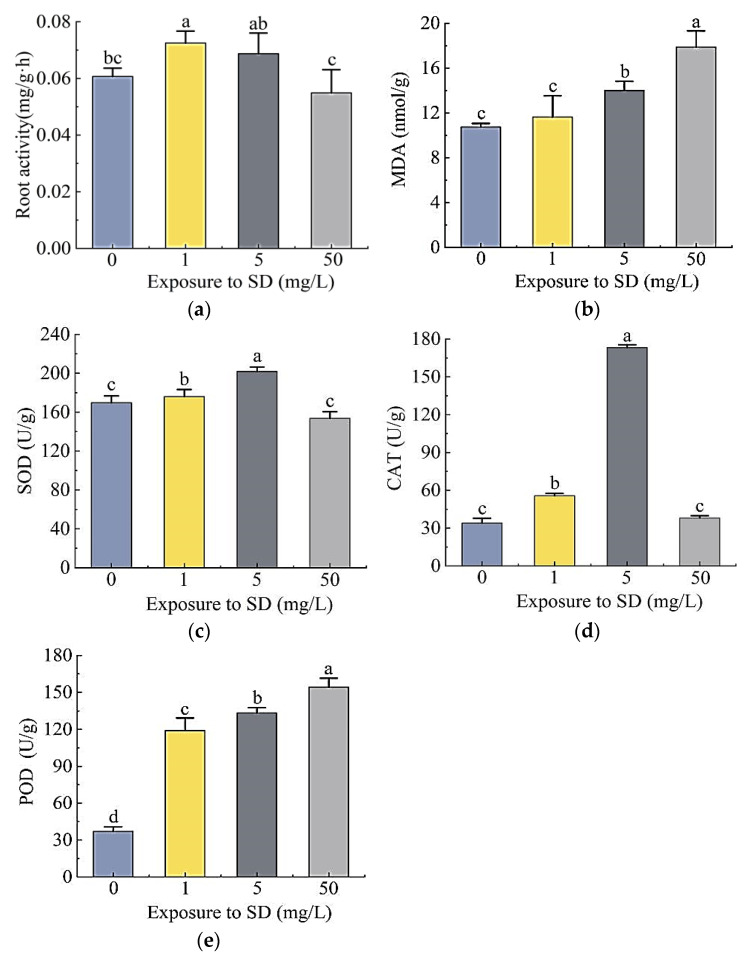
Effects of different concentrations of SD (0, 1, 5, and 50 mg/L) on root activity (**a**), leaf MDA (**b**), SOD (**c**), CAT (**d**), and POD (**e**) activities of *S. portulacastrum*. Data are mean ± standard deviation (*n* = 6). Different letters above the bars represent significant differences based on Duncan’s Multiple Range test at *p* < 0.05 level.

The exposure to different concentrations of SD significantly affected the root activity of *S. portulacastrum* plants as well as activities of malondialdehyde (MDA), superoxide dismutase (SOD), peroxidase (POD), and catalase (CAT) (Figure 3). Compared with the control, the root activity of plants exposed to 1 mg/L and 5 mg/L SD increased by 19.5% and 13.3%, respectively, inferring that SD promoted root activity (Figure 3a). The root activity of plants treated with 50 mg/L SD decreased by 9.5%, suggesting an inhibitory effect. The content of MDA in leaves increased with the increased concentrations of SD (Figure 3b). Compared with the control, leaf MDA concentrations in plants exposed to 1, 5, and 500 mg/L SD increased 8.4%, 30.5%, and 44.5%, respectively. The activities of SOD in plants exposed to 1 mg/L and 5 mg/L SD were 3.7% and 19.0% higher than that of the control, respectively; while for the 50 mg/L SD treatment, SOD decreased by 9.4% (Figure 3c). In reference to the control, CAT activities of plants treated with 1 mg/L and 5 mg/L SD increased 0.64 times and 4.1 times, respectively, while the CAT activity in 50 mg/L SD treatment was not significantly different from that of the control (Figure 3d). POD activity was the lowest in the control, while that in the treatment group increased 2.2, 2.6, and 3.2 times, respectively (Figure 3e).

### 2.3. The Impact of SD on Microbial Communities

**Figure 4:**
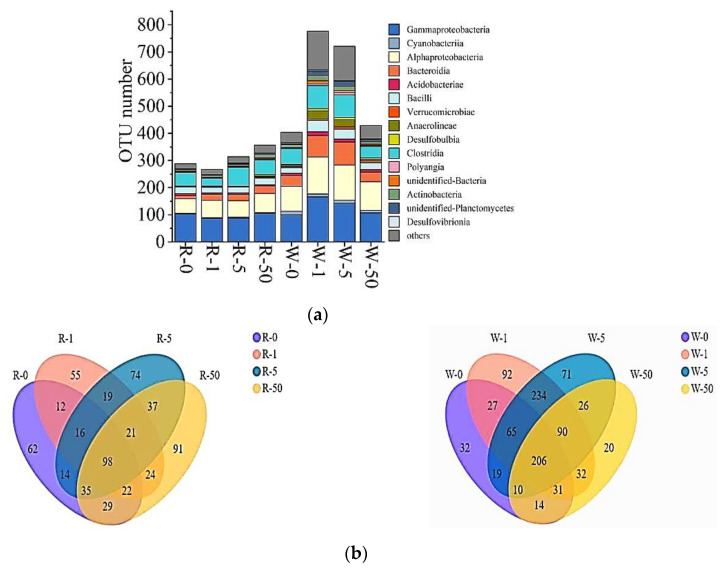
Relative abundance of bacterial groups (class level) based on DNA-derived approaches for each treatment (**a**) and Venn diagrams (**b**). The Venn diagrams show the numbers of OTUs (97% sequence identity) among treatments (R-0, R-1, R-5, and R-50 as rhizosphere samples collected from 0 mg/L, 1 mg/L, 5 mg/L, and 50 mg/L SD treatments, respectively; while W-0, W-1, W-5, and W-50 as water samples, collected from 0 mg/L, 1 mg/L, 5 mg/L, and 50 mg/L SD treatments, respectively).

In this study, a total of 105,185 bacterial sequences were detected, of which 57,687 high-quality sequences were obtained after splicing, quality control, and chimera removal. The high-quality sequences were clustered into operational taxonomic units (OTUs) based on 97% sequence similarity, and a total of 1025 OTUs were obtained. The microbial diversity in water and rhizosphere per treatment was analyzed (Figure 4a), and it was found that the overall microbial diversity in the water samples was higher than that of the rhizosphere samples. The order of microbial diversity in water was 771 OTUs (1 mg/L SD) > 721 OTUs (5 mg/L SD) > 429 OTUs (50 mg/L SD) > 404 OTUs (control). Whereas the order of rhizosphere microbial diversity was 357 OTUs (50 mg/L SD) > 314 OTUs (5 mg/L SD) > 288 OTUs (control) > 267 OTUs (1 mg/L SD). The Ace, Shannon, and Chao 1 diversity indexes also showed the same trend (Table 1). Regardless of water and rhizosphere, the main microbial groups were Gammaproteobacteria, Alphaproteobacteria, Clostridia, and Bacteroidia.

A total of 98 OTUs were shared by bacteria in the rhizosphere of *S. portulacastrum*, and the unique OTU of each treatment was 91 OTUs (50 mg/L SD) > 74 OTUs (5 mg/L SD) > 62 OTUs (control) > 55 OTUs (1 mg/L SD) (Figure 4b). The common OTUs of bacteria in the water samples were 206, and the unique OTU of each treatment group was 92 OTUs (1 mg/L SD) > 71 OTUs (5 mg/L SD) > 32 OTUs (control) > 20 OTUs (50 mg/L SD) (Figure 4b).

### 2.4. The Influence of SD on Microbial Compositions and Metabolic Activities

**Figure 5:**
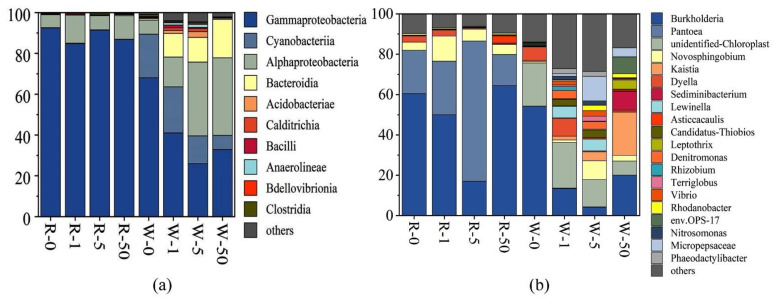
Microbial population dynamics in different treatments. Relative abundance of the top 10 classes (**a**). Relative abundance of the top 20 genera (**b**).

Based on the results of DNA sequencing, we determined the relative abundance of bacterial communities at the class and genus levels. As mentioned above, the dominant classes in the water samples were Gammaproteobacteria, Alphaproteobacteria, Cyanobacteria, and Bacteroidia. The dominant classes in plant rhizosphere were Gammaproteobacteria and Alphaproteobacteria (Figure 5a). The results showed that SD reduced the relative abundance of Gammaproteobacteria in the water samples. The relative abundance of Gammaproteobacteria in the control was 68.0%, but it was 41.0%, 26.0%, and 32.9% in 1 mg/L, 5 mg/L, and 50 mg/L SD treatments, respectively. However, SD increased the relative abundance of Bacteroidia in the water samples. Compared with the control, the relative abundance of Bacteroidia increased 13.3, 13.9, and 22.2 times in 1 mg/L, 5 mg/L, and 50 mg/L SD treatments, respectively, while the relative abundance of Gammaproteobacteria and Alphaproteobacteria in the rhizosphere samples did not change significantly. At the genus level, Burkholderia was the most dominant genus in both water and the rhizosphere samples, but the relative abundance of Burkholderia in the rhizosphere samples was significantly higher than that in the water samples (Figure 5b). The genus Pantoea had an absolute predominance in the rhizosphere samples, with a relative abundance ranging from 15.4% to 69.5%.

**Figure 6:**
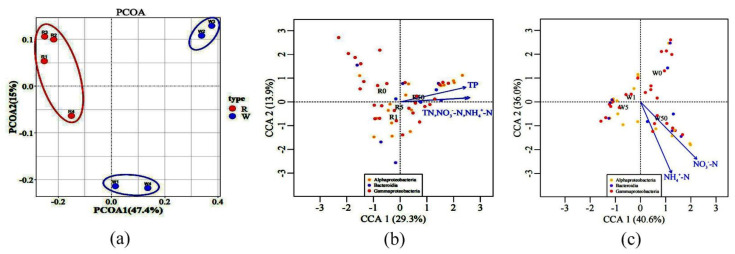
Principal coordinate analysis (PCoA) of bacteria in all treatments based on the Bray–Curtis distance matrix (**a**). Plot of the canonical correlation analysis (CCA) integrating environmental factors (TN: total nitrogen; NO_3_^−^-N: nitrate nitrogen; NH_4_^+^-N: ammonia nitrogen; and TP: total phosphorus) and metabolic activities of the bacterial groups, including Alphaproteobacteria, Bacteroidia, and Gammaproteobacteria. Only environmental factors with significant correlation (*p* < 0.05) are shown in the figure, including the rhizosphere (**b**) and water samples (**c**).

Principal coordinates analysis (PCoA) showed that microbial communities in water treated with 1 mg/L and 5 mg/L SD were different from that of the control, while the 50 mg/L SD treatment showed no significant difference from the control (Figure 6a). The differences in rhizosphere microbial communities between the control and the treatment groups were negligible. Subsequently, three main microbial groups, namely Gammaproteobacteria, Alphaproteobacteria, and Bacteroides, were selected. An analysis of the correlation between microorganisms and environmental factors using the canonical correlation analysis (CCA) revealed that microorganisms in the water samples were mainly affected by NH_4_^+^-N and NO_3_^−^-N, while the rhizosphere microorganisms were regulated by TP, TN, NH_4_^+^-N, and NO_3_^−^-N (Figure 6b,c).

## 3. Discussion

Nitrogen in water bodies exists in inorganic and organic forms, and P exists in the form of phosphate, polyphosphate, and organic phosphorus. Phytoremediation of N from polluted water relies primarily on plant uptake and the metabolic process of rhizosphere microorganisms, while the remediation of P is largely by means of root adsorption [21,22]. In this study, the presence of SD inhibits the growth of plants to a certain extent and affects the changes in microbial communities in water and rhizosphere and also in the plant–microbe interactions, which in turn adversely affects plant absorption of N and P and the microbial removal of N [23].

### 3.1. SD Effects on Plant Growth and Physiological Activities 

The fresh weights of *S. portulacastrum* exposed to SD were significantly reduced (Figure 2a). When the SD concentration reached 50 mg/L, the root numbers and their activity decreased significantly (Figure 2b and Figure 3a). Researchers have reported adverse effects on the growth and biomass of crack willow (*Salix fragilis* L.) and corn (*Zea mays* L.) by SD at a concentration of 200 mg/kg [24]. Moreover, antibiotics can also inhibit the growth of plant roots and destroy their structure [25]. These results suggest that SD inhibited plant growth; specifically, the inhibition of root growth would affect the plant’s ability to take up N and P. Our results also showed that SD had toxic effects on *S. portulacastrum*. When plants are subjected to SD stress, they produce a high amount of reactive oxygen species (ROS), which are eliminated by the cell’s antioxidant mechanism, thereby changing the antioxidant enzyme system [26,27]. Among them, SOD, POD, and CAT are some of the most important protective enzymes of the antioxidant defense system, which can eliminate certain ROS, maintain oxygen balance, and alleviate damage to the cell membrane [28,29]. SOD can turn O_2_- into H_2_O_2_; POD and CAT can cause further reduction of H_2_O_2_ [30,31]. The activities of SOD, POD, and CAT increased significantly in the 1 mg/L and 5 mg/L SD treatments (Figure 3c–e). Other studies have also showed that most antioxidant enzyme activities increase under stress conditions [32,33]. However, when the SD concentration at 50 mg/L, the activities of SOD and CAT in *S. portulacastrum* did not change significantly. This might be due to the fact that the content of O_2_- and H_2_O_2_ in the plant exceeded the scavenging ability of SOD and CAT, and the protective effect of antioxidant enzymes on plant cell membranes had certain limits. This finding was similar to that observed by Xu et al. [34]. When the soil SD concentration exceeded 25 mg/kg, the activity of plant antioxidant enzyme systems (SOD, POD, CAT) decreased [34]. In addition, MDA increased with increase in SD concentrations (Figure 3b). MDA is a toxic metabolism of lipid peroxidation. It can be used as an indicator of ROS generation and the degree of cell membrane damage, and its content can reflect the degree of stress in plants [30]. Therefore, our results indicated that the antioxidant capacity of plants was not enough to resist the increase in active oxygen concentrations when exposed to higher concentrations of SD. Studies have shown that increased MDA content will damage the photosynthesis process in plants [35]. Thus, the accumulation of MDA in *S. portulacastrum* might affect plant growth and the plant’s ability to remove N and P. After *S. portulacastrum* plants were exposed to SD for 21 days, the N content of different forms in the water body increased (Figure 1a–c), which might be due to cell membrane damages and root rot, resulting in the release of organic matter into the media and increased N content in the water body and deterioration of water quality [36,37].

### 3.2. SD Effects on Microbial Communities

In addition to plant uptake of N and P, microorganisms can also improve plant uptake of N and P through decomposition and transformation. The widespread existence of SD in water bodies may have a significant impact on the microbial community. Lin et al. reported that the microbial diversity increased when antibiotics were applied to the soil [38]. The results of this study suggested that with the increase of SD concentrations, the microbial diversity in the water body increased significantly (Figure 4). The groups with the most obvious change in the number of OTUs were Gammaproteobacteria, Alphaproteobacteria, and Bacteroides. They were the dominant classes in the water samples (Figure 5a) and were also the common microbial taxa in the samples that were heavily polluted with antibiotics [39]. There is a negative correlation between Gammaproteobacteria and sulfonamides, tetracyclines, and other antibiotics [40]. Our data also showed that the presence of SD reduced the relative abundance of Gammaproteobacteria in water (Figure 5a). Studies have indicated that both Gammaproteobacteria and Alphaproteobacteria are related to the nitrification process in biological denitrification [41,42]. As a result, the decrease in the relative abundance of Gammaproteobacteria could affect biological nitrification processes. Among the Gammaproteobacteria, the greatest change was Burkholderiales, which was considerably reduced. It has been reported that Burkholderiales is related to the nitrogen recycle [43]. As a result, the decrease in the relative abundance of Burkholderiales may affect the biological denitrification capacity.

The relative abundance of *Pseudomonas* within Gammaproteobacteria increased. This is probably attributed to the adaptability of *Pseudomonas* to antibiotics [44,45]. *Pseudomonas* has polyphosphate-accumulating properties, and the removal of phosphorus in water was less affected by SD. Additionally, the relative abundance of *Bacteroides* in the water samples increased significantly, and they were also resistant to antibiotics. Liao et al. reported that *Bacteroidia* contain bacterial genus that can degrade antibiotics [46]. In the water samples, *Burkholderia* and *Pantoea* were the main dominant genus. Relevant studies have revealed that this group could promote plant growth and resist diseases [47,48]. SD significantly reduced its relative abundance (Figure 5b), which might affect plant growth, thereby affecting the uptake of N and P. 

### 3.3. SD Effects on Plant–Microbe Interactions

The community structure of rhizosphere microorganisms was different from that of water samples (Figure 6a). SD had little effect on the microbial community structure in the rhizosphere. This is likely due to the root exudates in the rhizosphere that might be more conducive to stabilizing microorganisms. Previous studies have also proved that when plants are under stress, the positive impact of roots on microorganisms will form an effective community with high stress tolerance [49,50]; thus, the composition of the rhizosphere microbial community tends to be more stable. The relative abundance of Gammaproteobacteria in the rhizosphere microorganisms of *S. portulacastrum* was significantly higher than that of microorganisms in water (Figure 5a). This result was consistent with previous studies on the structure of the microbial community in the rhizosphere of *S. portulacastrum* where the roots of *S. portulacastrum* were enriched by Gammaproteobacteria compared to the non-rhizosphere [51]. However, the composition of the rhizosphere microbial community is impacted by a variety of biological and non-biological factors. Environmental conditions, such as N and P content, affect the rhizosphere microbial community [52]. The main groups of microorganisms in the water samples and rhizosphere samples, namely Gammaproteobacteria, Alphaproteobacteria, and Bacteroides, and their metabolic activity were regulated by N and P (TN, NH_4_^+^-N, NO_3_^−^-N, and TP) (Figure 6b,c). Some plants selected the microbial community according to the N and P in the environment to form a specific niche in the rhizosphere [53,54]. This led to differences between the water and root rhizosphere microbial community structures. These factors promote the changes in the plant rhizosphere microbial community, which may have an impact on the interaction system between the plant and the rhizosphere microbial community.

## 4. Materials and Methods

### 4.1. Experimental Materials

#### 4.1.1. Preparation of Different Concentrations of SD in Aquaculture Wastewater

SD (C_10_H_10_N_4_O_2_S, purity ≥ 98%) was purchased from Macklin Biochemical Co., Ltd. (Shanghai, China). In order to simulate the environment as well as to predict the effect of high concentrations of SD on plant growth, 50 mg/L of SD suspension was prepared as a stock solution. In reference to a study conducted on phytoremediation of sulfonamides [23], three SD concentrations were chosen by diluting the stock solution to 1×, 10×, and 50×, respectively, in the wastewater. The experimental wastewater samples were taken from an aquaculture farm in Fujian Province, China (119° E–26° N). The initial chemical concentrations in the aquaculture wastewater were as follows: TN, 24.8 ± 0.83 mg/L; TP, 4.3 ± 0.08 mg/L; NO_3_^−^-N, 5.2 ± 0.99 mg/L; and NH_4_^+^-N, 24.8 ± 0.06 mg/L. The pH was about 7.4, and the water temperature was 24.8 °C.

#### 4.1.2. Preparation of Plant Material

Stem cuttings of *S. portulacastrum* were provided by the Environmental Ecological Restoration Group of the Institute of Oceanography Minjiang University. The cuttings were grown in 1/4 Hoagland nutrient solution [55] at a temperature of 24 °C, with 16 h of light under a light intensity of 820 μmol/m^2^/s. After 10 d of cultivation, the cuttings were rooted with vigorous growth, and rooted cuttings with uniform overall growth were selected for the experiment.

### 4.2. Experimental Method

The selected rooted cuttings were planted in 3 L glass cylinder containers filled with the wastewater supplemented with SD at 0, 1, 5, and 50 mg/SD, respectively [23]. There were four plants per container, and three containers per treatment. The experiment was arranged as completely randomized design with three replications. Plants were grown under aforementioned conditions for 28 days. Water samples (150 mL) were collected every 7 days, and the fresh weight and root number of the plants were recorded. After 28 d of exposure, a part of the leaves was dried to constant weight and plant weight and moisture content were determined; the remaining leaves were frozen and stored at −80 °C in liquid nitrogen for measuring the plant enzyme activities. The plant roots were used to determine root activity. The remaining water samples and microorganisms attached to the root surface of the plant were analyzed for microbial community structure.

#### 4.2.1. Collection of Nutrient Salt Samples and Analysis Methods

The water samples collected in the experiment were immediately filtered through a 0.45 μm glass fiber membrane, and the nutrient salts were measured within 24 h. In this study, the nutrient salt indicators measured included TN, NH_4_^+^-N, NO_3_^−^-N, and TP. The nutrient salts analysis method was referred to the Standard Methods for Monitoring Water and Wastewater (4th edition) [56].

#### 4.2.2. Determination of Antioxidant Enzymes and Malondialdehyde in Plant Leaves and Root Activity

After 28 d of exposure, leaf samples were collected to determine enzyme activities (SOD, POD, CAT, MDA). Approximately 0.1 g of fresh leaves were taken, placed in a mortar, and cut to small pieces. Liquid nitrogen was added to the mortar, and the leaf pieces were quickly ground into a powder. The powder was transferred to centrifuge tube, after adding 0.9 mL of phosphate buffer (phosphate buffer: 0.1 mol/L pH = 7.4), the tube was vortexed for 3 min and centrifuged at 4 °C for 10 min at 2500× *g*, and the supernatant (10% of the homogenate supernatant) was used for further study. The operation steps were followed strictly, according to the kit instructions; the kits were purchased from Jiancheng Bioengineering Institute (Nanjing, China). Plant roots (0.2–0.5 g) were washed and blotted with filter paper. The root activity was measured through the triphenyl-tetrazolium chloride (TTC) method. The operation steps were followed strictly, according to the kit instructions; the kit was purchased from BestBio Co. Ltd. (Shanghai, China).

#### 4.2.3. Collection of Water Samples and High-Throughput Sequencing 

Plant roots were cut and placed in 2 L of sterile sodium chloride solution and centrifuged for 4 h at 2500× *g* to obtain water samples containing rhizosphere microorganisms. The rhizosphere water sample and remaining wastewater samples were filtered through a 200 μm silk-screen to remove impurities and larger biological debris. The microorganisms in the wastewater and rhizosphere were enriched on 0.2 μm nucleic acid fiber membranes (142 mm diameter, Millipore, Burlington, MA, USA) by a pump. The microbial-enriched fiber membranes were stored in sterile centrifuge tubes at −80 °C, and high-throughput sequencing was performed by Novogene (Nanjing, China). PCR amplification of the V4 region of 16S rRNA genes was performed, and the following primers were used: upstream primer 515F (5′GTGCCAGCMGCCGCGGTAA-3′) and downstream primer 806R (5′GGACTACHVGGGTWTCTAAT-3′). After PCR amplification, the PCR products were purified and sequenced through the Illumina NovaSeq high-throughput sequencing platform (Nanjing, China).

### 4.3. Data Analysis and Statistics

All data were subjected to analysis of variance using SPSS 21.0 (IBM Corp, Armonk, NY, USA). If significant differences occurred, means were separated by Duncan’s Multiple Range test at *p* < 0.05 level. Data were presented as mean ± standard deviation (SD). Plotted images were generated using Origin 2018.

The original sequence after splicing was referred as per Qiime [57] (V1.9.1 http://qiime.org/scripts/split_libraries_fastq.html (accessed on 15 September 2021) for strict quality control. The sequence to detect and remove chimeras after quality control passes the UCHIME algorithm (UCHIME Algorithm, http://www.drive5.com/usearch/manual/uchime_algo.html (accessed on 15 September 2021) [58] to obtain high-quality sequences. Finally, the high-quality sequences of each sample were clustered by OTUs with 97% consistency, and the sequences of OTUs were compared by the Mothur method and the SSUrRNA database of SILVA 138 [59,60]. When analyzing OTUs that appeared only in one sample, those that contained only one sequence and those that were annotated as non-bacterial species were removed. In order to avoid errors caused by different sequencing depths between samples, all DNA sequencing results in this study were uniformized with the smallest number of sequences in the sample. All statistical analyses in this study were performed with R language software (V.4.0.5) [61]. PCoA was used to evaluate the differences between different microbial samples.

## 5. Conclusions

Eutrophication represents a serious challenge in the management of water bodies. Phytoremediation is an environmentally benign technology for reducing N and P in contaminated water. However, such remediation of N and P could be substantially hampered by the presence of antibiotics in the contaminated water. The present study showed that SD adversely affected the growth of *S. portulacastrum*. With increased concentrations of SD, fresh weight and root numbers decreased, antioxidant enzyme activities increased, and the removal of N and P was reduced. SD changed the structure of the microbial community in the water as the relative abundance of Burkholderiales related to N removal significantly decreased, and the abundance of *Burkholderia* and *Pantoea* that could promote plant growth was significantly reduced, thereby affecting N and P removal. Additionally, the community structure of microorganisms in water and rhizosphere was different. Compared with water, the microbial community structure in rhizosphere was less affected by SD. The differences might influence the interaction between plants and rhizosphere microbial community and affect the removal of N and P, but such interactions require further investigation.

## Figures and Tables

**Table 1:** Bacterial diversity in different samples analyzed using Ace, Shannon, and Chao 1 diversity.

Sample/Index		ACE	Shannon	Chao 1
R	R-0	340.07	1.56	332.38
	R-1	304.30	1.62	297.69
	R-5	370.86	1.24	357.56
	R-50	421.08	1.57	416.38
W	W-0	465.31	1.95	441.67
	W-1	842.95	3.73	846.00
	W-5	739.91	4.02	727.62
	W-50	516.71	2.99	519.02

Note: R and W represent rhizosphere and water samples, respectively. R-0, R-1, R-5, and R-50 are rhizosphere samples collected from 0 mg/L, 1 mg/L, 5 mg/L, and 50 mg/L SD treatments, respectively; while W-0, W-1, W-5, and W-50 are water samples, collected from 0 mg/L, 1 mg/L, 5 mg/L, and 50 mg/L SD treatments, respectively.

## Data Availability

The data presented in this study are available in the article.
